# Involvement of nucleophosmin/B23 in TPA-induced megakaryocytic differentiation of K562 cells

**DOI:** 10.1038/sj.bjc.6601100

**Published:** 2003-09-30

**Authors:** C Y Hsu, B Y M Yung

**Affiliations:** 1 Graduate Institute of Pharmacology, National Yang Ming University, Taiwan, Republic of China; 2 Genomics Research Center & Cancer Biochemistry Laboratory, Department of Pharmacology, College of Medicine, Chang Gung University, 259 Wen-Hwa 1st Road, Kwei-San, Tao-Yuan 333, Taiwan, Republic of China

**Keywords:** nucleophosmin/B23, TPA, megakaryocytic differentiation

## Abstract

Human myelogenous leukaemia K562 cells were induced to undergo megakaryocytic differentiation by treatment with phorbol ester 12-*O*-tetradecanoylphorbol-13-acetate (TPA) (20 nM, 24–72 h). The steady-state level of nucleophosmin/B23 mRNA decreased during the TPA-induced differentiation. There was also decrease in the level of cellular nucleophosmin/B23 protein and appearance of its degraded product (25 kDa) during the TPA-induced differentiation. Furthermore, K562/B23 (wild type), K562/D1 (Δ280–294) and K562/D2 (Δ263–294) cells were less, while K562/D3 (Δ244–294) cells were more responsive to TPA-induced differentiation as compared to K562/vector or parental K562 cells. Activation of the ERK/MAPK was observed in parental K562 cells upon TPA treatment (5 nM, 5–30 min). As compared to K562/vector cells, less activation of ERK/MAPK was observed in K562/D2 cells, while ERK/MAPK was highly activated in K562/D3 cells upon TPA treatment. Our results indicate that nucleophosmin/B23 plays an important role in TPA-induced differentiation of K562 cells and the amino acids 244–294 at C-terminal of nucleophosmin/B23 could be an important site for regulation of cellular response to differentiation.

A coordination and balance between cell proliferation, differentiation and apoptosis is crucial for normal development and tissue-size homeostasis in the adult (Li and Yuan, 1999). Dysregulation of this tightly controlled mechanism of cell differentiation and apoptosis may result in cancer. Mutated cell survival and inappropriate proliferation, disrupting this balance, would be expected to contribute to oncogenesis (Wyllie, 1997). Tumour cells often acquire damage to genes that directly regulate cell growth to result in uncontrolled cell proliferation. The identification of genes and their products that are involved in response to growth stimuli is essential for understanding of the normal cell growth and its regulation (Wolff, 1997).

Between cancer and normal cells, one important difference is the hyperactivity and the pleomorphism of the nucleoli in cancer cells (Busch *et al*, 1963; Busch, 1990). The nucleolus includes many proteins such as enzymes for RNA synthesis (RNA polymerase I) and processing (exonuclease and endonuclease), the proteins of preribosomes and nonribosomal proteins (Prestayko *et al*, 1974). Recent reports have shown that nonribosomal proteins of the nucleolus participate in regulating nucleolar activity that is associated with cell growth (Valdez *et al*, 1994). Of the proteins found in elevated amounts in nucleoli of cancer cells, a nonribosomal protein nucleophosmin/B23 is present in one of the largest amounts on two-dimensional gel electrophoresis (Prestayko *et al*, 1974; Orrick *et al* 1973). Nucleophosmin/B23, being more abundant in tumour cells than in normal resting cells, has a potential role in increased nucleolar activity that is necessary for cell proliferation (Feuerstein *et al*, 1988; Chan *et al*, 1989); a role as a cytoplasmic/nuclear shuttle protein (Borer *et al*, 1989); relieves transcription repression by YY1 (Inouye and Seto, 1994); binds nuclear and nucleolar localisation signals on the HIV Type 1 Rev protein (Fankhauser *et al*, 1991) and the human T-cell leukaemia virus-1-Rex protein (Adachi *et al*, 1993); binds cell cycle-regulated nucleolar protein p120 (Valdez *et al*, 1994); inhibits DNA-binding and transcriptional activity of interferon regulatory factor-1 (IRF-1), which is a tumour suppressor (Tanaka *et al*, 1994; Kondo *et al*, 1997). Nucleophosmin/B23 overexpression at the RNA and protein levels may contribute to the onset of cancer (Feuerstein *et al*, 1988; Chan *et al*, 1989; Kondo *et al*, 1997). Blockage of nucleophomin/B23 expression with its antisense oligonucleotides has shown that nucleophosmin/B23 is crucial for making cancer cells resistant to induction of differentiation and apoptosis (Hsu and Yung, 1998; Liu and Yung, 1998; You *et al*, 1999). It thus appears that an excess of nucleophosmin/B23 is an important cause of cancer and not just a consequence.

The K562 cell line was originally established from the pleural effusion of a patient with chronic myelogenous leukemia (CML) in the blastic phase (Tabilio *et al*, 1983). The K562 cell line can be induced to differentiate towards megakaryocytic differentiation by the phorbol ester 12-*O*-tetradecanoylphorbol-13-acetate (TPA) and resulted in loss of proliferative capacity (Alitalo, 1990). 12-*O*-tetradecanoylphorbol-13-acetate likely activates pathways of signal transduction regulating cell proliferation and differentiation. K562 cell line becomes an important tool for the study of early events involved in megakaryocytic differentiation. The aim of our present study was to investigate the role of nucleophosmin/B23 in megakaryocytic differentiation. Attempt was made to elucidate the effect of C-terminal deletion of nucleophosmin/B23 on TPA-induced differentiation of K562 cells. Our results demonstrate that nucleophosmin/B23 plays a role in the control of cellular response to TPA-induced megakaryocytic differentiation of K562 cells.

## MATERIALS AND METHODS

### Drugs and antibodies

12-*O*-tetradecanoylphorbol-13-acetate was purchased from Sigma Chemical Co. (St Louis, MO, USA). Monoclonal anti-phospho-ERK and anti-ERK antibodies (Abs) were from Santa Cruz (Santa Cruz, CA, USA). Monoclonal anti-CD41a-FITC and anti-CD42b-FITC Abs were purchased from BD Pharmingen (San Diego, CA, USA). Antiactin, anti-FLAG and fluorescein-conjugated goat anti-mouse IgG Abs were from Sigma. Monoclonal Ab to nucleophosmin/B23 was kindly provided by Dr PK Chan, Department of Pharmacology, Baylor College of Medicine, Houston, TX, USA.

### Cell culture

K562 leukaemia cells were grown in RPMI-1640 medium (Life Technologies, Rockville, MD, USA) supplemented with 10% heat-inactivated foetal bovine serum (Hyclone, UT, USA), 2 mM glutamine, 50 U ml^−1^ penicillin and 50 *μ*g ml^−1^ streptomycin in a 5% humidified incubator at 37°C. K562 cells were treated with TPA (80 nM). Every 24 h for 4 days, cultures were harvested and monitored for cell number by counting cell suspensions with haemocytometer. Cell viability was assessed by exclusion of 0.2% trypan blue.

### Assays of cellular differentiation

TPA-induced megakaryocytic differentiation of K562 cells was assayed by measuring the expressions of differentiation-specific membrane antigens (CD41a and CD42b). Cells were washed and suspended in ice-cold PBS containing 10% foetal calf serum. Anti-CD41a-FITC or anti-CD42b-FITC Ab was added for 30 min at 4°C and the cells were then washed twice with 2 ml of PBS containing 10% foetal calf serum. The percentages of CD41a- or CD42b-positive cells and fluorescence intensities were evaluated by FACScan. The threshold value discriminating positive from negative cells was set as the fluorescence level below which 95% of control (TPA untreated) cells occurred.

### Immunoblot analysis

Cells were harvested, washed twice in ice-cold PBS and lysed in RIPA buffer (1% Triton X-100, 1% sodium deoxycholate, 0.1% SDS, 20 mM Na_2_HPO_4_, 100 mM NaCl, 20 mM NaF, 0.2 mM PMSF, 1 mM DTT, 30 *μ*g ml^−1^ DNase and 30 *μ*g ml^−1^ RNase). The lysates were boiled in SDS sample buffer (62.5 mM Tris, pH 6.8, 5% *β*-mercaptoethanol, 10% glycerol, 2% SDS, 0.001% bromophenol blue) and the cell extract was fractionated by 10% SDS–polyacrylamide gel electrophoresis (SDS–PAGE). The separated proteins in SDS–PAGE were electrotransferred to Hybond-PVDF membrane (Amersham Pharmacia). The PVDF membrane was then soaked in a blocking solution (5% nonfat milk in TBST buffer (20 mM Tris-HCl, pH 7.5, 0.5 M NaCl, 0.1% Tween 20)) for 1 h at room temperature. The soaked PVDF membrane was then incubated with primary antibody overnight at 4^o^C, washed with TBST buffer three times for 15 min each and incubated at room temperature for 1 h in horseradish peroxidase-conjugated goat anti-mouse IgG Ab (Promega, Madison, WI, USA). The membrane was washed with TBST buffer three times for 15 min each. Immunoreactivity was determined using the chromogenic development or the enhanced chemiluminescence reaction (ECL, Amersham).

### RNA analysis

Total RNA was prepared from K562 cells by ULTRASPEC™ RNA Isolation System (Biotecx, Houston, TX, USA). For Northern blot analysis, aliquots of 5 *μ*g of RNA were separated by 1.2% formaldehyde agarose gel electrophoresis, transferred to nylon membranes (Amersham) by downward alkaline capillary method and fixed to the membrane by drying at 80°C for 30 min. The nucleophosmin/B23 cDNA labelled with [*α*-^32^P]dCTP using a random primed kit (Promega, Madison, WI, USA) was employed as a probe for detection of homologous mRNA. Prehybridisation was carried out overnight at 42°C in a solution containing 50% formamide, Denhardt's solution, 5 × SSC (1 × SSC=0.15 M NaCl, 0.015 M sodium citrate), 0.1% SDS, and 250 *μ*g ml^−1^ denatured salmon sperm DNA. Radiolabelled probe at a specific activity of 1–2 × 10^8^ c.p.m. *μ*l^−1^ was hybridised with total RNA in the same solution for 24 h at 42°C. Washing of the membranes for the probes was twice in 2 × SSC and 0.5% SDS at 42°C for 10 min, and once in 0.1 × SSC and 0.5% SDS at room temperature for 30 min. The radioactive nucleophosmin/B23 homologous mRNA was determined by autoradiography with phosphoimager or with intensifying screen at −70°C.

### Plasmid

Full-length nucleophosmin/B23 cDNA in plasmid PET-T7 (which was generously given by Dr Pui Kwong Chan, Department of Pharmacology, Baylor College of Medicine, Houston, TX, USA) was amplified by PCR using 5′-GCG TGC CGC CAC CCG ATG GAA GAT TCG ATG G-3′ and 5′- GTT TAA ACT ATT TTC TTA AAG AGA CTT-3′ as primers. Amplified PCR products were then separated and isolated from 1% agarose gel. The 0.9 kilobase nucleophosmin/B23 cDNA was then subcloned into the cloning site of the vector pCR™3 supplied in the Eukaryotic TA cloning kit (Invitrogene, Carlsbad, CA, USA). The orientation of the cDNA in pCR™3 was determined by nucleotide sequencing using the Sequence kit (Amersham Pharmacia Biotech, Buckinghamshire, England, UK). The plasmid clones containing the nucleophosmin/B23 cDNA in the sense orientation in respect to the cytomegalovirus (CMV) immediate-early promoter of pCR™3 was referred as pCR3-B23.

For full-length nucleophosmin/B23 cDNA clones (T7), the N-terminal primer was 5′- ACC ATG GAC TAC AAA GAC GAT GAC GAC AAG CTT ATG GAA GAT TCG ATG GAC-3′. This primer encoded an AUG translation initiation codon followed by the codons for the eight amino acids in the FLAG epitope (Asp-Tyr-Lys-Asp-Asp-Asp-Asp-Lys) and six amino acids from nucleophosmin/B23. The C-terminal primer that contained the *Bam*HI site was 5′-CGC CGC GGA TCC TTA AAG AGA CTT CCT CCA CT-3′. For carboxyl-terminal deletion mutants, the N-terminal primer was the same as T7 clone as described above. C-terminal primers were up to deletion sites and stop codon (TAA) was added in the terminus. The C-terminal primers for deletion mutants were: D1, 5′-GCG CCT AGG TTA AGT CAT CCG GAA GC-3′ (Δ280–294); D2, 5′-GCG CCT AGG TTA GGG AAG AGA ACC ACC-3′ (Δ263–294); D3, 5′-GCG CCT AGG TTA AGA ACT AGG TCC-3′ (Δ244–294). Plasmid pET-T7 nucleophosmin/B23 cDNA was used as a template. After PCR amplification, the FLAG tag and the coding region of nucleophosmin/B23 should be in frame. The PCR products were cloned into pCR3.1 vector supplied in Eukaryotic TA cloning kit (Invitrogene, Carlsbad, CA, USA). The orientation of each construct was analysed by restriction mapping and full-length nucleotide sequencing using the Sequence kit (Amersham Pharmacia Biotech). The plasmid clones containing the full-length nucleophosmin/B23 or deletion mutant cDNA were driven by CMV immediate-early promoter.

### Establishment of stable clones

The transfection was performed using Lipofectamine™ Reagent (Life Technologies) method. K562 cells were seeded at a density of 5 × 10^5^ per well in 1.0 ml serum-free medium. A measure of 2.5 *μ*g of pCR3.1-FLAG-B23, pCR3.1-FLAG-D1, pCR3.1-FLAG-D2, pCR3.1-FLAG-D3 plasmid constructs or pCR3 vector alone and 12.5 *μ*g of lipofectamine reagents in serum-free medium were mixed gently. The mixture was incubated for 30 min at room temperature, added to K562 cells and was incubated overnight at 37°C in the CO_2_ incubator. The culture was further incubated in fresh RPMI-1640 medium for 48 h. The transfected cells were then distributed in 24-well plates at 500 cells per well, and 800 *μ*g ml^−1^ G418 was added for selection of stably transfected clones. After selection with G418 for 3–5 weeks, individual clones were expanded to mass cultures and subsequently assayed for nucleophosmin/B23 or its deletion mutant expression. The transfectants were maintained in culture medium supplemented with 200 *μ*g ml^−1^ of G418. Clones overexpressing nucleophosmin/B23 or its deletion mutants were screened by Western blot using monoclonal anti-FLAG Ab.

## RESULTS

### Decrease of nucleophosmin/B23 mRNA during TPA-induced differentiation of K562 cells

**Figure 1:**
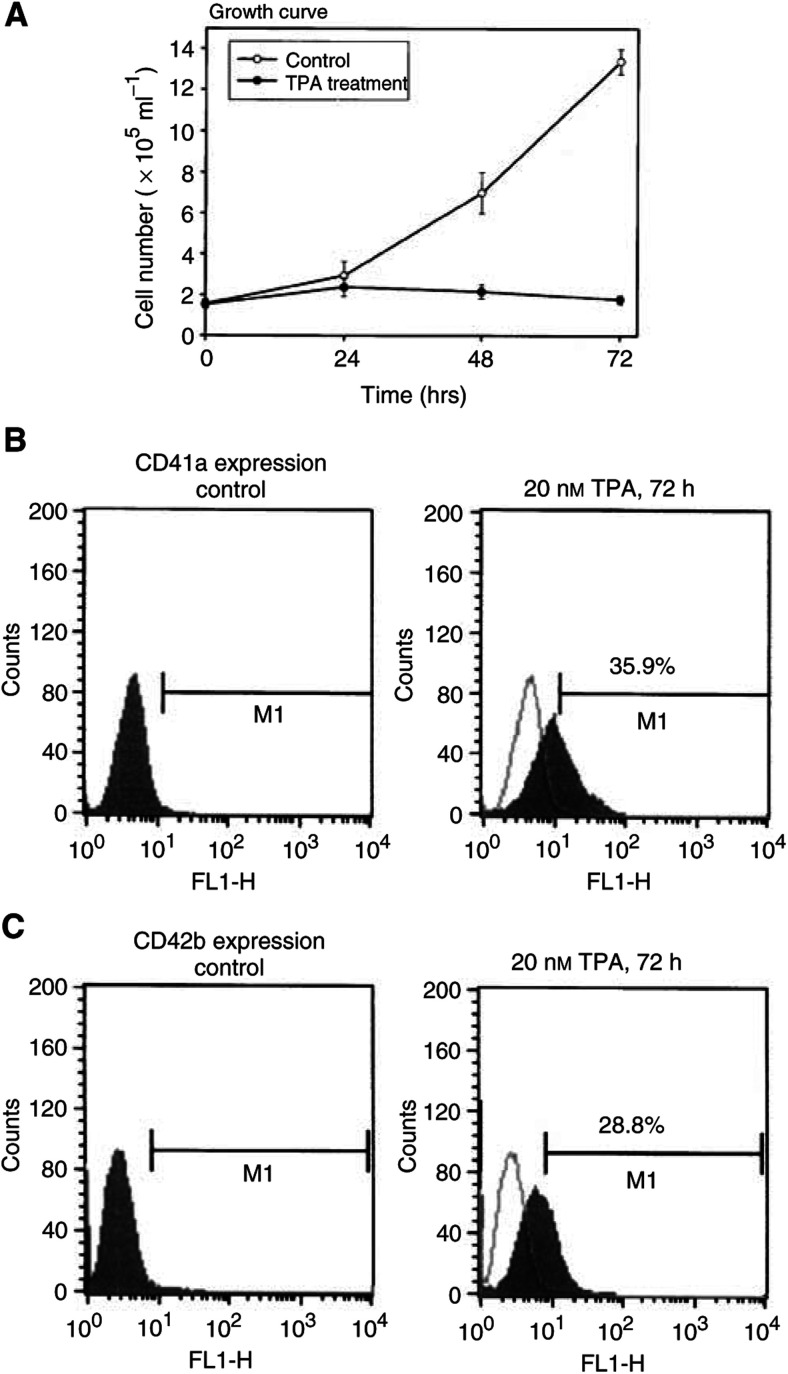
Effect of TPA on cell growth and differentiation of K562 cells. (**A**) The growth curve after 20 nM TPA treatment for various times (24–72 h). Cultures were harvested and cell numbers were counted with a haemocytometer. Points, means of triplicate±SD. Viability of the cells was >90% under these TPA treatments. (**B**,**C**) K562 cells were treated with 20 nM TPA for 72 h and the differentiation was assayed by measuring the expressions of differentiation markers. Expressions of the CD41a or CD42b were evaluated by flow cytometry using specific anti-CD41a or anti-CD42b FITC-conjugated monoclonal antibody.

**Figure 2:**
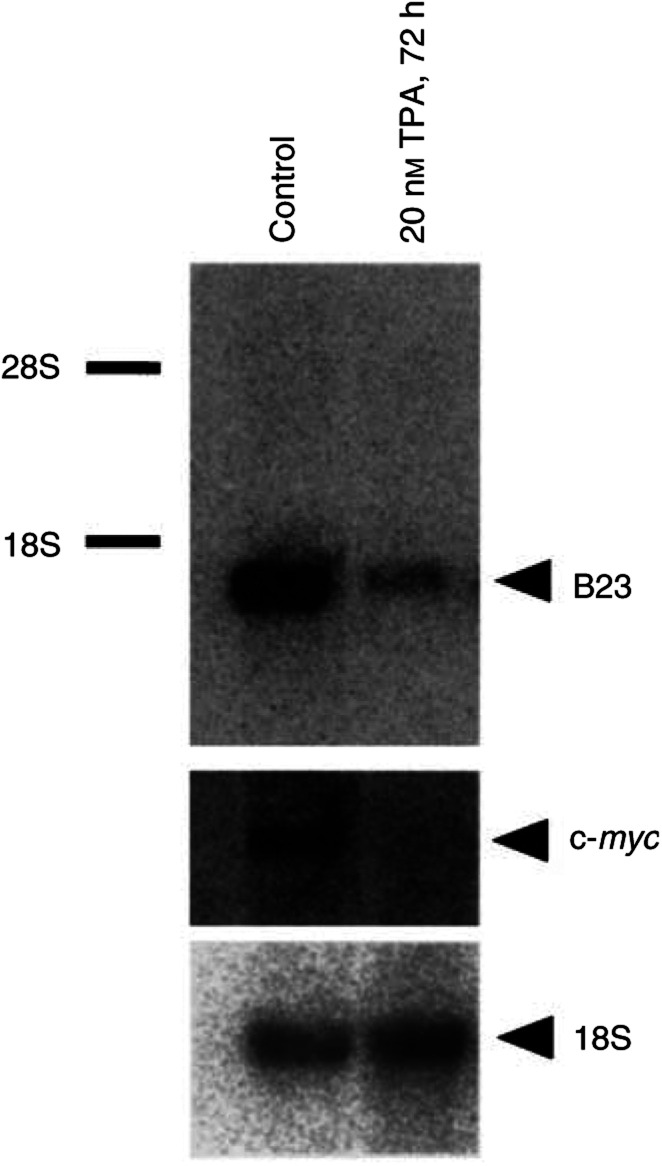
Decrease of nucleophosmin/B23 mRNA during TPA-induced differentiation of K562 cells. K562 cells were treated with 20 nM TPA for 72 h. Cells were then harvested and the total RNA was prepared. Northern blot analysis was performed with 5 *μ*g of RNA for each sample. The ^32^P-labelled nucleophosmin/B23 (B23) cDNA and c-*myc* probes were employed for detection of homologous mRNA. The same filter was hybridised with ^32^P-labelled 18S cDNA probe that was used as a control for the amount of RNA loaded.

To investigate whether nucleophosmin/B23 was involved in megakaryocytic differentiation, we first examined nucleophosmin/B23 mRNA expression during TPA-induced differentiation of human myelogenous leukaemia K562 cells. K562 cells can be induced to differentiate towards megakaryocytic lineage, characterised by changes in cell morphology, cell growth arrest and acquisition of megakaryocytic surface markers (Braverman *et al*, 1986; Alitalo, 1990). Culturing the cells for 24–72 h with 20 nM TPA caused growth inhibition (Figure 1A

) and a marked increase in differentiation surface marker, CD41a or CD42b (Figure 1B, C). The viability of the cells was over 90% as measured by trypan blue method over 72 h of 20 nM TPA treatment (data not shown). A single mRNA band hybridizing with nucleophosmin/B23 cDNA probe was observed in Northern blot of rapidly proliferating leukaemic K562 cells (Figure 2

). After 72 h of 20 nM TPA treatment, nucleophosmin/B23 mRNA expression decreased to less than 20%. In parallel, c-*myc* mRNA expression was also decreased during TPA-induced differentiation (Figure 2).

### Decrease of nucleophosmin/B23 protein level during TPA-induced differentiation of K562 cells

**Figure 3:**
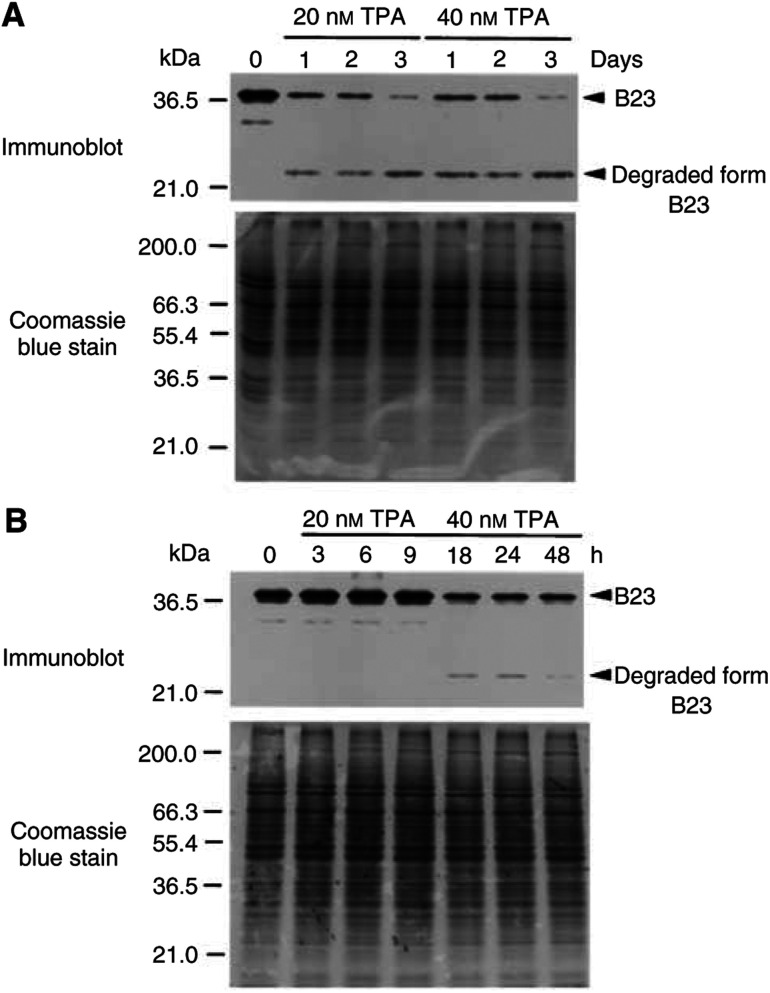
Decrease of nucleophosmin/B23 protein level during TPA-induced differentiation of K562 cells. (**A**) K562 cells were treated with 20 or 40 nM TPA for various times (1–3 days) or (**B**) treated with 20 nM TPA for various times (3–48 h). Cells were harvested and 10 *μ*g of total cellular proteins was separated by 10% SDS–PAGE stained with Coomassie blue (lower panels) or blotted onto PVDF membrane (upper panels). Nucleophosmin/B23 (B23) protein was detected by Immunoblot using anti-nucleophosmin/B23 monoclonal Ab and the chromogenic development.

Total cellular protein samples (containing equal amounts of protein) from control untreated K562 cells and the K562 cells treated with 20 or 40 nM TPA for various times (1–3 days; 3–48 h) were separated by 10% SDS–PAGE and subsequently analysed by Western blot immunoassay. The lower and upper panels of Figure 3A or B

showed the Coomassie blue-stained SDS–PAGE and chromogenic diagrams of Western blot analysis, respectively. After 1–3 days of TPA (20–40 nM) treatment, the Western blot showed that the cellular protein level of nucleophosmin/B23 decreased and a new band at 25 kDa appeared (Figure 3A). The appearance of 25 kDa band was detected after 18 h of 20 nM TPA treatment (Figure 3B). Caspase-3 inhibitor (25 *μ*M; Ac-DEVD-CHO) blocked the decrease of nucleophosmin/B23 and the appearance of the new band at 25 kDa that could possibly be the degraded form of nucleophosmin/B23 (data not shown).

### Establishment of nucleophosmin/B23 wild-type and deletion mutants overexpressed stable clones

**Figure 4:**
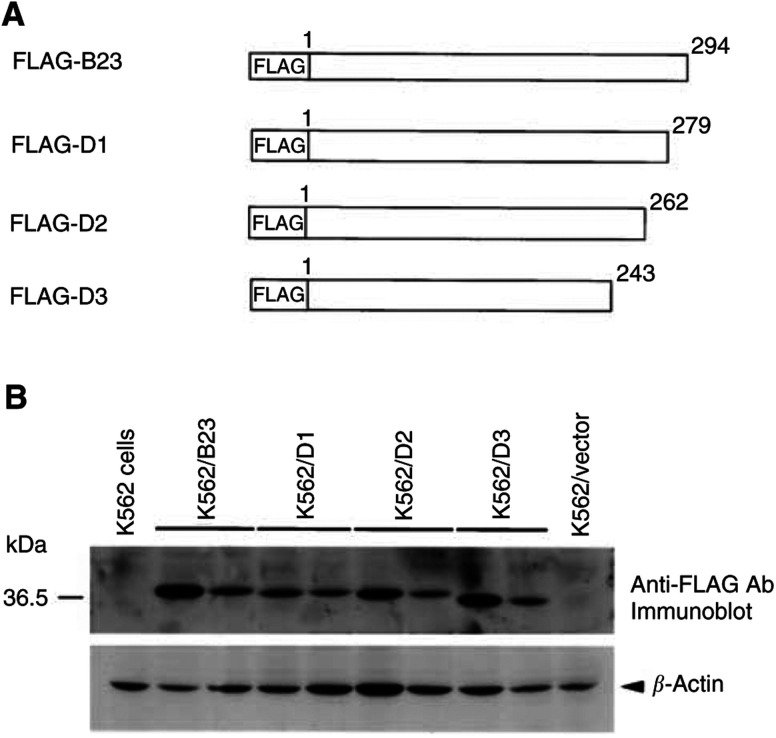
Schematic representation of nucleophosmin/B23 wild-type and deletion mutants. (**A**) The human nucleophosmin/B23 cDNA or its deletion mutants were cloned into the expression vectors pCR3.1 in frame with the amino terminus of the FLAG epitope (Asp-Tyr-Lys-Asp-Asp-Asp-Asp-Lys). The first and last amino acids residues are indicated by numbers. The open boxes represent the open reading frame of nucleophosmin/B23 wild-type and deletion mutants. (**B**) Immunoblot analysis of overexpression of nucleophosmin/B23 wild-type or deletion mutants in stable clones of K562 cells. Total protein was extracted from the parental K562 cells, the control vector-transfected K562 cells (K562/vector) and stable clones (two for each) of nucleophosmin/B23 wild-type (K562/B23) or deletion mutants (K562D1-D3). A measure of 50 *μ*g of total cellular proteins was separated by 10% SDS–PAGE and blotted onto PVDF membrane. FLAG-wild-type nucleophosmin/B23 (FLAG-B23), FLAG-D1, FLAG-D2, FLAG-D3 or *β*-actin protein was detected by Western blot using anti-FLAG or anti-*β*-actin monoclonal antibody. Immunoreactivity was determined by the ECL reaction.

Attempt was made to investigate which domain of nucleophosmin/B23 was involved in TPA-induced differentiation of K562 cells. K562 cells were transfected with nucleophosmin/B23 wild-type or deletion constructs fused each at its amino terminus to the FLAG epitope, encoding eight amino acids (Asp-Tyr-Lys-Asp-Asp-Asp-Asp-Lys) (Figure 4A

). After G418 selection, stable clones of K562 cells overexpressing nucleophosmin/B23 wild-type and various deletion constructs, namely K562/B23 (wild type), K562/D1 (Δ280–294), K562/D2 (Δ263–294) and K562/D3 (Δ244–294), were established. We obtained two clones each from various stable clones of nucleophosmin/B23 wild-type or deletion mutants. Western blot analysis with anti-FLAG antibody showed that nucleophosmin/B23 wild-type and deletion proteins were overexpressed in those stable clones (Figure 4B) and were distinguishable from cellular endogenous nucleophosmin/B23. The apparent mobility of the proteins corresponded well with the predicted molecular weight of each deletion mutant. The predicted sizes were as followed: FLAG-nucleophosmin/B23, 39 kDa; FLAG-D1, 36 kDa; FLAG-D2, 35 kDa; FLAG-D3, 33 kDa. K562/B23, K562/D1, K562/D2 and K562/D3 cells grew similarly and showed no morphological changes as compared with the control vector-transfected K562 cells (K562/vector) or the parental cells (K562 cells) (data not shown).

### Assessment of cellular response of nucleophosmin/B23 wild-type and deletion mutant overexpressed cells to TPA-induced differentiation

**Figure 5:**
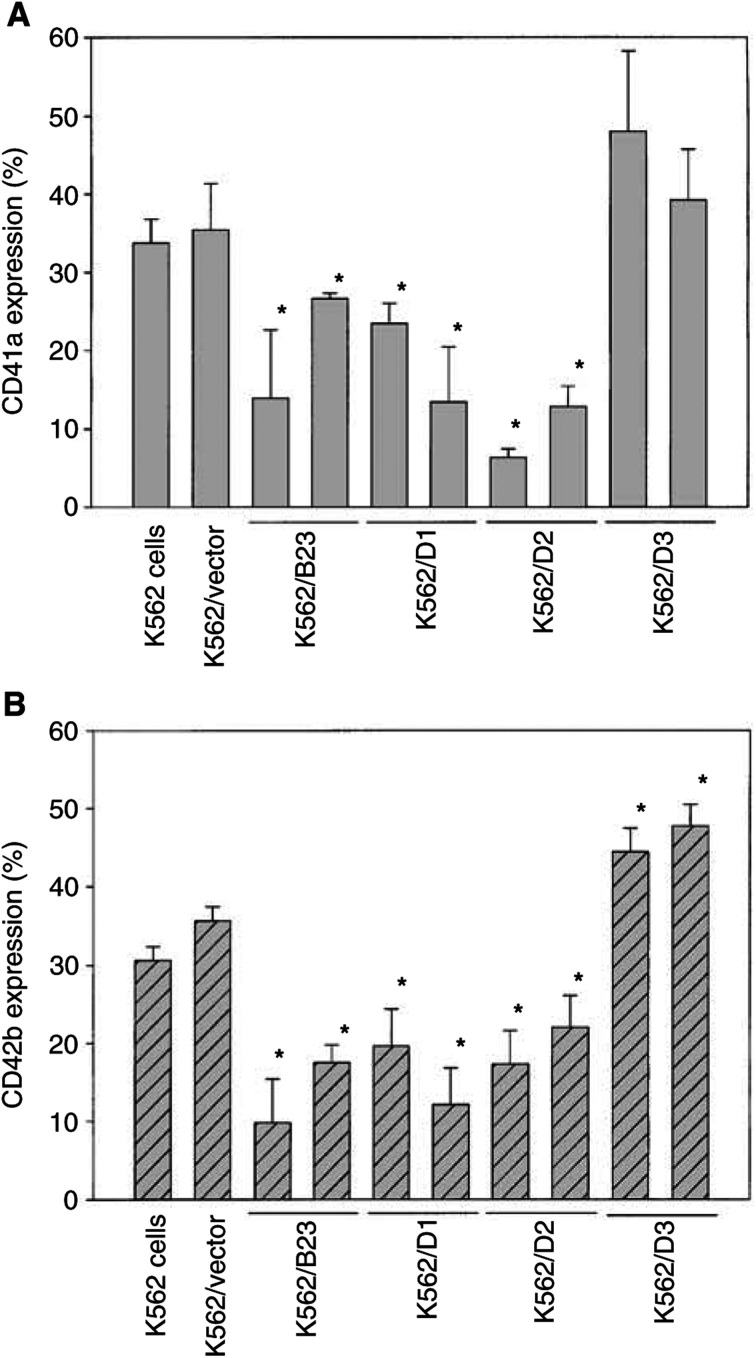
Assessment of cellular response of nucleophosmin/B23 wild-type and deletion mutant overexpressed cells to TPA-induced differentiation. The parental K562 cells, the control vector-transfected K562 cells (K562/vector) and the stable clones (two for each) of nucleophosmin/B23 wild-type (K562/B23) or deletion mutants (K562/D1, K562/D2 or K562/D3) were treated with 20 nM TPA for 72 h. After the cells were harvested, expressions of CD41a (**A**) or CD42b (**B**) were evaluated by flow cytometry using specific anti-CD41a or anti-CD42b FITC-conjugated monoclonal antibody. Bars, means of triplicate±SD. ^*^P<0.05, as compared with K562/vector cells under same TPA treatment.

Flow cytometric analysis of surface markers showed that expression of CD41a or CD42b was lower in K562/B23 (wild type), K562/D1 (Δ280–294) or K562/D2 (Δ263–294) cells as compared to control vector-transfected cells (K562/vector) or the parental cells (K562 cells) treated with 20 nM TPA for 72 h (Figure 5

). In contrast, expression of CD42b was higher in K562/D3 (Δ244–294) cells as compared to K562/vector or parental K562 cells treated with 20 nM TPA for 72 h (Figure 5). Our results showed that K562/B23 (wild type), K562/D1 (Δ280–294) and K562/D2 (Δ263–294) cells were less, while K562/D3 (Δ244 – 294) cells were more responsive to TPA-induced differentiation as compared to K562/vector or parental K562 cells. These results indicated that amino acids 244–294 of the nucleophosmin/B23 protein could be an important site for regulation of cellular response to differentiation. It is possible that deletion of C-terminal (amino acids 244–262) of nucleophosmin/B23 leads to the loss of its binding ability with some regulatory factor(s). The factor(s) being free from complexing with nucleophosmin/B23 is then functional for induction of differentiation.

### Activation of ERK upon TPA treatment

**Figure 6:**
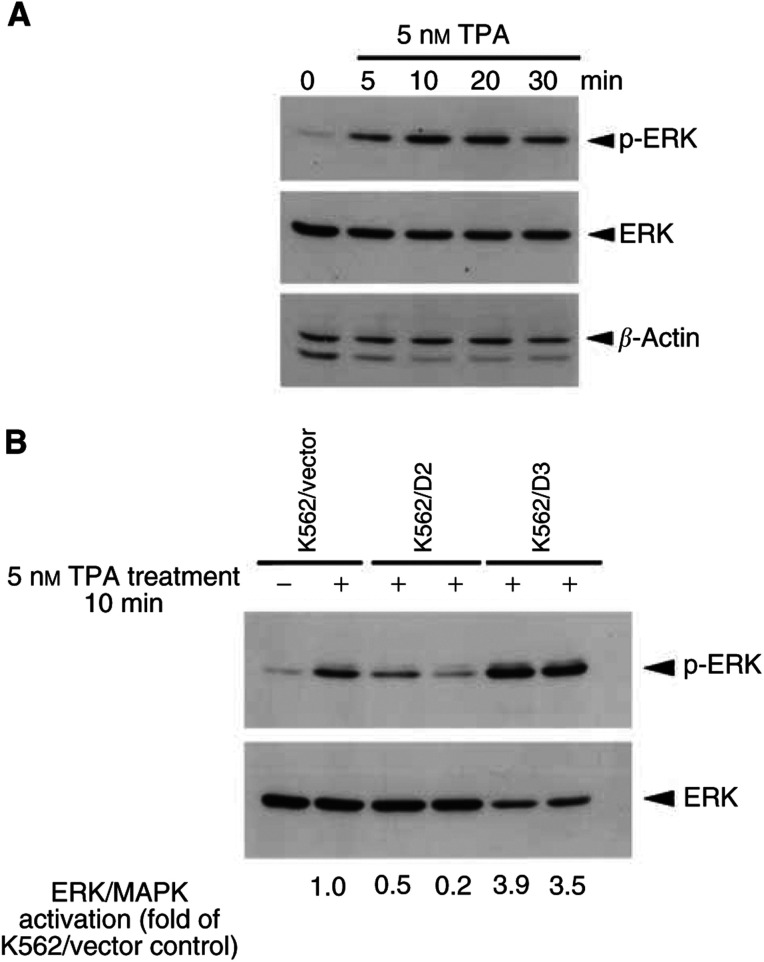
Immunoblot analysis of activated ERK (p-ERK) or total ERK level upon TPA treatment. (**A**) K562 cells were treated with 5 nM TPA for various times (5–30 min). (**B**) K562/D2, K562/D3 or the control K562/vector cells (two lanes for each stable clone) were treated with 5 nM TPA for 10 min. Cells were harvested and 20 *μ*g of total cellular proteins was separated by 10% SDS–PAGE and blotted onto PVDF membrane. p-ERK, ERK (total) or *β*-actin protein were detected by Western blot using anti-p-ERK, ERK or *β*-actin monoclonal antibody and the ECL reaction.

Previous studies have shown that ERK/MAPK is activated during megakaryocytic differentiation of K562 cells (Racke *et al*, 1997; Whalen *et al*, 1997). To elucidate whether nucleophosmin/B23 was involved with ERK/MAPK in TPA-induced differentiation, we examined the effect of TPA on ERK activation in nucleophosmin/B23 deletion mutant overexpressed K562/D2 (less responsive to TPA) and K562/D3 (more responsive to TPA) cells as compared to K562/vector cells (Figure 6

). Activation of the ERK/MAPK was observed in parental K562 cells upon TPA treatment (5 nM, 5–30 min) (Figure 6A). As compared to K562/vector cells, less activation of ERK/MAPK was observed in K562/D2 cells, while ERK/MAPK was highly activated in K562/D3 cells upon TPA treatment (5 nM, 10 min) (Figure 6B).

## DISCUSSION

This study investigated the possibility that nucleophosmin/B23 played an important role in cellular susceptibility to TPA-induced megakaryocytic differentiation of K562 cells. We demonstrate that nucleophosmin/B23 mRNA and protein are decreased after TPA treatment in parental K562 cells. Stable clones of K562 cells overexpressing nucleophosmin/B23 wild-type and various deletion constructs, namely K562/B23 (wild type), K562/D1 (Δ280–294), K562/D2 (Δ263–294) and K562/D3 (Δ244–294) have been established to investigate which domain of nucleophosmin/B23 is involved in TPA-induced differentiation of K562 cells. Our results have shown that K562/B23 (wild type), K562/D1 (Δ280–294) and K562/D2 (Δ263–294) cells are less, while K562/D3 (Δ244–294) cells are more responsive to TPA-induced differentiation as compared to K562/vector or parental K562 cells. These results indicate that amino acids 244–294 of the nucleophosmin/B23 protein may be an important site for regulation of cellular response to TPA-induced differentiation of K562 cells. C-terminal end has been shown to be important site of nucleophosmin/B23. DNA polymeraseα binding site is at the C-terminal. A total of 12 amino acids at the C-terminal end of B23.1, which are absent in B23.2, is essential for full stimulation of the DNA polymerase *α* (Umekawa *et al*, 2001). Deletion of C-terminal (amino acids 244–262) of nucleophosmin/B23 may lead to the loss of its binding ability with important growth regulatory factor(s). Stimulation of growth activity was inhibited or factor(s) being free from complexing with nucleophosmin/B23 is then functional for induction of differentiation.

In RA-induced differentiation of HL-60 cells, the involvement of nucleophosmin/B23 in oncogenic activity is that nucleophosmin/B23 may bind and inactivate the tumour suppressor in cancer cells (Hsu and Yung, 2000). IRF-1 acts as a transcriptional activator in the interferon system and as a tumour suppressor (Harada *et al*, 1989,^1993^). Nucleophosmin/B23 could bind to IRF-1, interfere with IRF-1 binding to IRF-1 response elements, inhibit the IRF-1 transcriptional activity and manifest oncogenic potential (Kondo *et al*, 1997; Hsu and Yung, 2000). Interaction of nucleophosmin/B23 with some factor(s), such as tumour suppressor, may be an important mechanism in the control of cellular response to induction of cellular differentiation and apoptosis. The availability of the binding site of nucleophosmin/B23 to interact with tumour suppressor is the basis for the cells being abnormal and resistant to induction of differentiation and apoptosis. In the present study, little induction of IRF-1 protein was observed in TPA-induced differentiation of K562 cells. All GST-nucleophosmin/B23 and the deletion mutants (D1-D3) could bind the *in vitro* translated ^35^S IRF-1 (our unpublished data). Nucleophosmin/B23 may act through other factors rather than IRF-1 in the control of TPA-induced differentiation in K562 cells.

Decrease of nucleophosmin/B23 has been observed in cells during induction of cellular differentiation and apoptosis (Hsu and Yung, 1998; Liu and Yung 1998). More drastic decrease of nucleophosmin/B23 is detected in NIH-3T3 than in ras-transformed cells during the apoptosis induced by serum deprivation (Chou and Yung, 2001). Nucleophosmin/B23 in serum-deprived NIH-3T3 cells is found to be highly unstable, with a half-life of less than 4 h. Cell-permeable caspase-3 inhibitor (10–25 *μ*M) blocks the decrease of nucleophosmin/B23 induced by serum deprivation in NIH-3T3 cells (Chou and Yung, 2001). These studies indicate that increased stability of nucleophosmin/B23 is involved in antiapoptosis. While no evidence of cleavage of nucleophosmin/B23 under apoptotic or necrotic conditions was found in HL-60 cells before (Bortul *et al*, 2001), the appearance of its degraded forms has now been detected in serum-deprived NIH-3T3 (Chou and Yung, 2001) and TPA-treated K562 cells (the present study). Signalling pathway and cell specificity involved with the cleavage of nucleophosmin/B23 in the induction of apoptosis and differentiation need further investigation.

Mitogen-activated protein kinase (MAPK) modules are involved in the signal transduction of a wide variety of signals in the eukaryotic organisms. The ERK/MAPK cascade plays a pivotal role in several cellular functions. The ERK/MAPK is activated by dual phosphorylation on a threonine and a tyrosine residue, achieved by the dual-specificity kinase MAP kinase kinase (MEK) (Waskiewicz and Cooper, 1995). In our present study, activation of the ERK/MAPK is observed in parental K562 cells upon TPA treatment. As compared to K562/vector cells, less activation of ERK/MAPK is observed in K562/D2 cells, while ERK/MAPK is highly activated in K562/D3 cells upon TPA treatment. Our results indicate that nucleophosmin/B23 plays a role in cellular response to ERK/MAPK-activated megakaryocytic differentiation of K562 cells.

Nucleolus participates in many other aspects of gene expression as well (Pederson, 1998). Biosyntheses of signal recognition particle RNA and telomerase RNA involve a nucleolar stage (Pederson, 1998) and nucleolus is a site critical to cellular aging (Johnson *et al*, 1998). Nucleolar protein nucleophosmin/B23 is importantly associated with cancer (You *et al*, 1999) and is implicated to have a functional role in the apoptotic cascade (Patterson *et al*., 1995) and growth control (Hsu and Yung, 1998; Liu and Yung, 1998). The potentiation ability of nucleophosmin/B23 antisense in induced cellular differentiation, apoptosis and inhibition of telomerase activity is particularly interesting and may lead to the use of antisense construct in cancer treatment. Taken together, the present study represents one of the few demonstrations of the involvement of a nuclear protein in the control of cell death/cell differentiation. The detailed mechanism or transduction cascade involved in nucleophosmin/B23-mediated resistance to induction of differentiation and apoptosis is under current investigation.

In conclusion, our results provide evidence that nucleophosmin/B23 plays an important role in TPA-induced megakaryocytic differentiation of K562 cells.
